# Phylogenetic Analysis Indicates a Longer Term Presence of the Globally Distributed H58 Haplotype of *Salmonella* Typhi in Southern India

**DOI:** 10.1093/cid/ciz1112

**Published:** 2020-01-31

**Authors:** Agila Kumari Pragasam, Derek Pickard, Vanessa Wong, Gordon Dougan, Gagandeep Kang, Andrew Thompson, Jacob John, Veeraraghavan Balaji, Ankur Mutreja

**Affiliations:** 1 Department of Clinical Microbiology, Christian Medical College, Vellore, India; 2 Department of Medicine, Univesity of Cambridge, Cambridge, United Kingdom; 3 Wellcome Sanger Institute, Hinxton, United Kingdom

**Keywords:** typhoid; H58, *Salmonella* Typhi, AMR, India

## Abstract

**Background:**

Typhoid fever caused by *Salmonella* Typhi is a major public health concern in low-/middle-income countries. A recent study of 1900 global *S*. Typhi indicated that South Asia might be the site of the original emergence of the most successful and hypervirulent clone belonging to the 4.3.1 genotype. However, this study had limited samples from India.

**Methods:**

We analyzed 194 clinical *S*. Typhi, temporal representatives from those isolated from blood and bone marrow cultures in southern India, over 26 years (1991–2016). Antimicrobial resistance (AMR) testing was performed for most common clinical agents. Whole-genome sequencing and SNP-level analysis was conducted. Comparative genomics of Vellore isolates was performed to infer transmission and AMR events.

**Results:**

We identified multidrug-resistance (MDR)–associated clade 4.3.1 as the dominant genotype. We detected 4.3.1 *S*. Typhi as early as 1991, the earliest to be reported form India, and the majority were fluoroquinolone resistant and not MDR. MDR was not detected at all in other genotypes circulating in Vellore. Comparison with global *S*. Typhi showed 2 Vellore subgroups (I and II) that were phylogenetically highly related to previously described South Asia (subgroup I, II) and Southeast Asia (subgroup II) clades.

**Conclusions:**

4.3.1 *S*. Typhi has dominated in Vellore for 2 decades. Our study would assist public health agencies in better tracking of transmission and persistence of this successful clade in India and globally. It informs clinicians of the AMR pattern of circulating clone, which would add confidence to their prophylactic/treatment decision making and facilitate efficient patient care.

## Teaser

The molecular epidemiology of *Salmonella* Typhi circulating in India since early 1990s and its dominance is reported. An in-depth AMR profiling of the major drug-resistant lineage, 4.3.1, would help in management and for public health agencies in planning intervention strategies.

Typhoid fever is a systemic infection caused by the gram-negative enteric bacteria *Salmonella enterica* serovar Typhi (*S*. Typhi) [1]. *Salmonella* Typhi is a human-restricted pathogen that persists as a serious public health challenge, with an estimated 20.6 million infections and approximately 220,000 deaths annually, predominantly in low- and middle-income countries (LMICs) [2–8]. The clinical symptoms of typhoid overlap with other febrile illnesses and diagnosis requires laboratory culture of the organism from blood or other clinical samples, presenting a challenge in lower-resource settings. Thus, the actual disease incidence is likely to be significantly underestimated. The challenges of managing typhoid have been compounded by the emergence of multidrug-resistant (MDR) *S*. Typhi, initially involving resistance to ampicillin, chloramphenicol, and trimethoprim-sulfamethoxazole, which were original first-line agents for treatment [9]. The emergence of MDR *S*. Typhi led to a change in empirical treatment regimens involving the use of fluoroquinolones such as ciprofloxacin [10, 11]. However, their excessive use has quickly led to a phenomenon of “decreased ciprofloxacin susceptibility” (DCS), driven in part by mutations in the quinolone resistance-determining region (or QRDR) within genes encoding DNA gyrase (*gyrA, gyrB*) and topoisomerases (*parC, parE*) [12].

Genomic and phylogenetic analysis of *S*. Typhi has shown that genetic variation is limited in this serovar and that all isolates originated from a common ancestor, which adapted to humans several thousand years ago [13]. Thus, to understand the rapidly changing behavior of this bacteria, it is essential to understand the epidemiological and evolutionary history of *S*. Typhi, including antimicrobial resistance (AMR), in different settings against a global background [14–16]. Such analysis has shown that a particular MDR *S*. Typhi clone, known as haplotype H58 or 4.3.1, has emerged over recent decades to dominate the epidemiological landscape in Asia and Africa [17]. The 4.3.1 genotype has displaced other *S*. Typhi lineages, and resistance in this lineage has involved the acquisition of IncHI1 plasmids, as well as QRDR-associated mutations [18].

Phylogenetic analysis indicated that South Asia might be the site of the original emergence of the 4.3.1 genotype [17], but this hypothesis was based on sequence data from a limited number of *S*. Typhi isolates from India and adjacent countries. Understanding the evolution and phylogenetic relationship of *S*. Typhi in the region is of critical importance to understand its origin. In this study, we sequenced the genomes of a collection of *S*. Typhi isolated from Vellore, a southern India region, and report the emergence of 4.3.1 from 1991 onwards. We also characterized AMR in these isolates at both the phenotypic and genotypic level to monitor trends and correlate genomic and phenotypic AMR.

## METHODS

### Bacterial Isolates

*Salmonella* Typhi isolates were from blood and bone marrow cultures obtained from individuals attending the Christian Medical College Hospital, Vellore, between 1991 and 2016. A total of 194 *S*. Typhi were retrieved from the archived stocks (4462 isolates) and selected for detailed genomic analysis. These 194 isolates were chosen as being representative of temporal distribution of this study period.

### Microbial Identification and Serotyping

All isolates were confirmed as *S*. Typhi using the classical biochemical tests for this serovar as per the standard laboratory protocols: serotyping was performed based on slide agglutination tests using antisera including polyvalent O antisera A-I, Vi, group D, and serovar specific antisera STO and STH (Becton Dickinson).

### Antimicrobial Susceptibility Testing

Susceptibility was determined for the following antimicrobials using disc tests with concentrations of antibiotics as follows: ampicillin (10 µg), chloramphenicol (30 µg), trimethoprim/sulfamethoxazole (1.25/23.75 µg), ceftriaxone (30 µg), azithromycin (15 µg), ciprofloxacin (5 µg), and pefloxacin (5 µg). Zone diameters were measured and interpreted as per Clinical Laboratory Standards Institute (CLSI) guidelines [18]. In addition, minimum inhibitory concentrations (MICs) were determined by E-test for ciprofloxacin and the results were interpreted as per CLSI guidelines [19].

### DNA Extraction

Whole-genomic DNA was extracted following overnight culture in Luria Bertanii broth incubated at 37°C. DNA was extracted using Wizard Genomic DNA kits (Promega) following the manufacturer’s instructions. The extracted DNA was then quantified using Nanodrop, Thermofischer Scientific.

### Whole-genome Sequencing and Phylogenetic Analysis 

Genomic libraries were prepared with unique indexing of each DNA sample, and up to 190 libraries were sequenced per lane of Illumina HiSeq V4 platform. Subsequently, 125-bp paired-end sequencing was performed and reads obtained were segregated using index tag information before mapping against the *S*. Typhi CT18 (accession number AL513382) reference genome. The study isolates were assigned to the previously described lineages based on the single nucleotide polymorphisms (SNPs) using an extended genotypic framework and genotyped as 4.3.1 and/or other genotypes [20]. Recombination hot spots and plasmids of CT18 were masked in the final alignment before calling SNPs between all the genomes using methods previously described [17]. Single nucleotide polymorphisms were further qualified by quality score to minimize errors, and any SNP that was not present in at least 75% of the reads was not included in the analysis. Any unmapped reads and the sequences that were not present in all the genomes were not taken forward for the phylogenetic analysis. RAxML version 0.7.4 [21] and the general time-reversible (GTR) model with gamma correction were used for drawing a consensus, bootstrapped phylogenetic tree based on the alignment of SNPs called from the whole genome. The tree was midpoint rooted with display arranged in increasing node order. For the 4.3.1 lineage subtrees, CT18 was used as an outgroup and root.

### Resistome Analysis From the Whole Genome

*Salmonella* Typhi genomic data were analyzed for the presence of resistance determinants including *bla*_*TEM*_, *cat*, *dfrA*-1, *sul1*, and *sul2* and the presence or absence of IncHI1 plasmids using in silico polymerase chain reaction (PCR) analysis. Mutations in the QRDR of gyrase (*gyrA, gyrB*) and topoisomerase (*parC, parE*) genes were analyzed for the DCS phenotype.

## RESULTS

### Genotypes of *Salmonella* Typhi Isolated in Vellore

Of the 4462 *S*. Typhi isolated during the study period (1991–2016), 194 that were representative of the temporal distribution were selected for more detailed genomic analysis. Among these, 13 different genotypes were identified, indicating a broad representation across the serovar phylogenetic population structure. Seventy-seven percent (n = 149) of the isolates belonged to the 4.3.1 genotype, indicating the dominance of this genotype in the population. The 4.3.1 genotype was present throughout 1991 to 2016, and its prevalence was already significant in 1991 (64%), the first year of the study period. The second largest clade was 2.5.0 (n = 15, 7.7%), which was one among the oldest genotypes identified previously from Indian isolates during 1977 [17], and was observed between 1991 and 2009. Four isolates belonged to a novel genotype, 3.2.0, which was not previously identified, even in the global collection of 1900 *S*. Typhi. All other genotypes were represented by fewer than 5 isolates, with the exception of 4.1.0 (n = 6, 3.0%), 3.2.1 (n = 5, 2.5%), and 2.2.2 (n = 5, 2.5%), followed by 2 isolates each of 2.4.0, 3.0.2, and 3.3.1 and 1 isolate each of 2.0.0, 2.2.1, and 3.3.0, respectively. One isolate, B22992 (2014), despite clustering within the 4.3.1 clade, was designated as 4.0.0 due to a deletion of 27 nucleotides covering a critical SNP typing region.

### Vellore *Salmonella* Typhi in a Global Phylogenetic Framework

**Figure 1.**
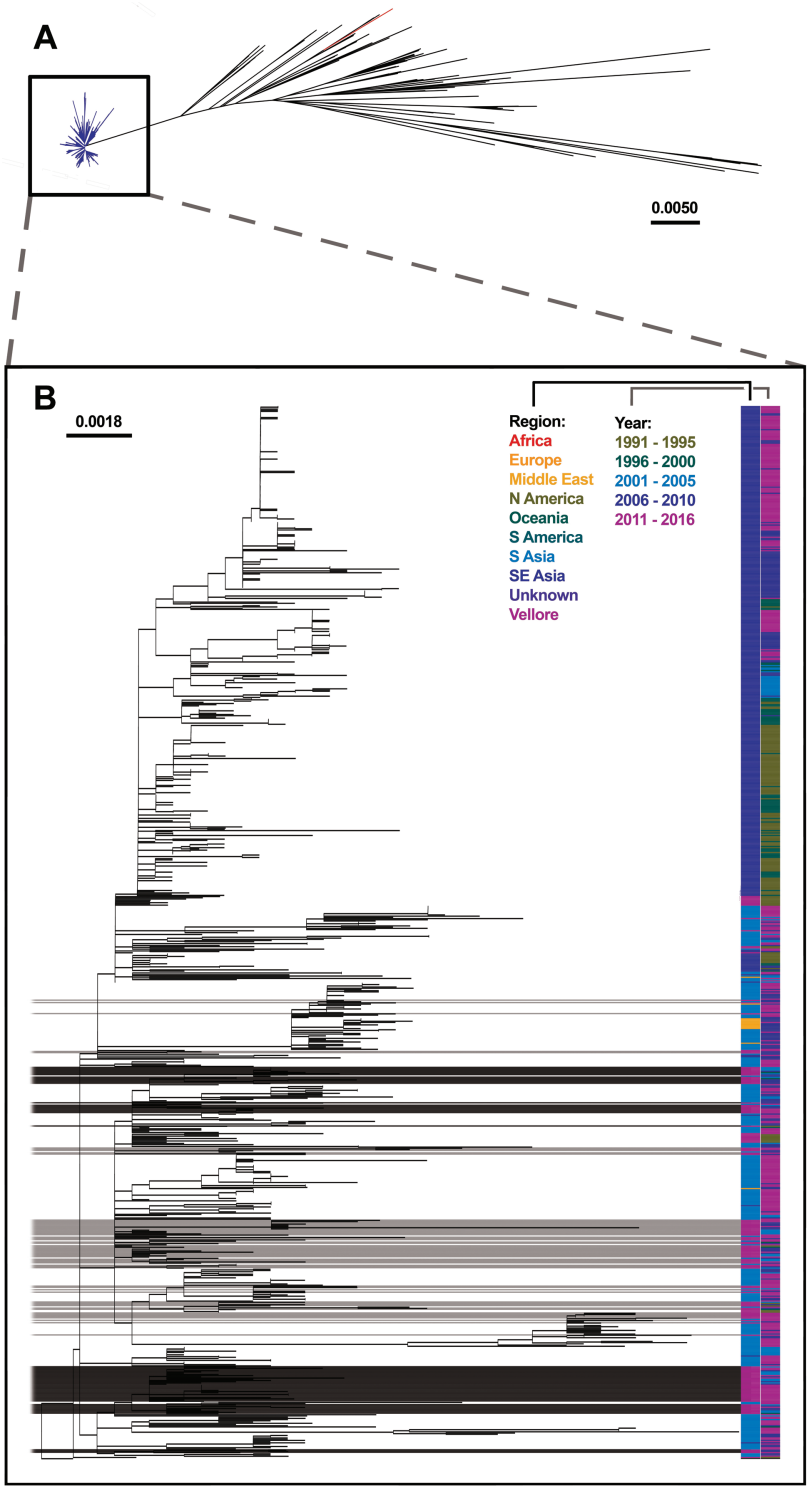
*A*, Maximum likelihood phylogenetic tree of 965 *Salmonella* Typhi genomes based on 6941 single nucleotide polymorphisms, mapped against the CT18 reference genome, indicating a clear overall distinction between 4.3.1 and other genotypes of *S*. Typhi. *B*, Representation of the clustering of the 4.3.1 lineage of Vellore isolates with global 4.3.1 at the phylogenetic level. Place and year of isolation are according to the color key. The scale bar indicates the number of substitutions per site. Genotype 4.3.1 of Vellore *S*. Typhi belonging to subgroups I and II are highlighted as dark (subgroup I) and light (subgroup II) gray streaks. Abbreviations: S, South; SE, Southeast.

A genomic comparison was made between the 194 sequenced *S*. Typhi from Vellore and a collection of 771 *S*. Typhi from 60 countries covering multiple genotypes (Figure 1, Supplementary Table 1). The maximum likelihood phylogenetic tree, based on SNPs, showed a clear distinction between 4.3.1 and other genotypes (Figure 1A), and the isolates from Vellore clustered as per their respective genotypes as expected. Within the global 4.3.1 lineage (Figure 1B), there was no tight clustering observed among the Vellore isolates. Instead, our study isolates clustered with previously sequenced South Asian and Southeast Asian isolates. These data provide compelling evidence of the frequent transmission of 4.3.1 isolates between South Asia and Southeast Asia. The earliest 4.3.1 *S*. Typhi in the entire global collection is from Vellore in 1991. Based on the evolution rate of 0.63 SNPs per genome per year [17], the most recent common ancestor (MRCA) of Vellore 4.3.1 and other genotypes was approximately 158 years ago. In Vellore, evolution rates of non–4.3.1 genotypes are comparatively higher, after divergence from their MRCA. After divergence from the first isolate of the 4.3.1 genotype, the mutation rates were slower. Thus, the lower mutation rates within 4.3.1 support fitness of the successful clone, with its stable genomic compatability and enhanced capability for spread and local clonal expansion.

**Figure 2.**
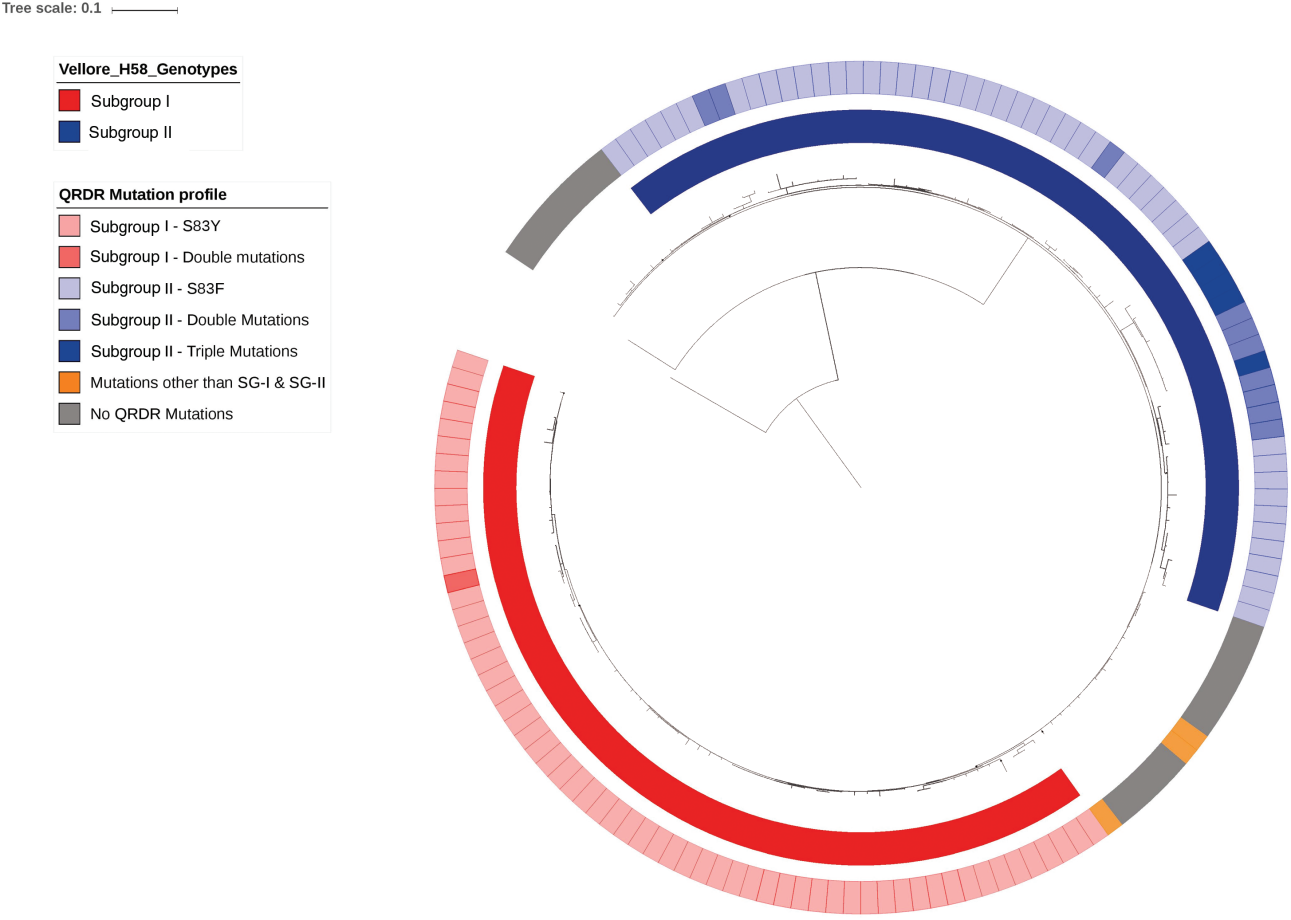
Maximum likelihood tree of 150 *Salmonella* Typhi genomes of the 4.3.1 genotype from Vellore, rooted against the CT18 genome, inferred from 916 single nucleotide polymorphisms. The innermost circle represents the 2 subgroups (SG-I, red; SG-II, blue). The outer circle denotes details of subgroups I and II with shades representing each subgroup. Blue shades of subgroup II indicate the presence of additional double and triple mutations, compared with subgroup I. Gray shading refers to the *S*. Typhi 4.3.1 genotype with no QRDR mutations. The scale bar indicates the number of substitutions per site. Abbreviations: QRDR, quinolone resistance-determining region; SG, subgroup.

Genomic analysis of *S*. Typhi isolates from Vellore indicates the evolution of 2 major 4.3.1 subgroups within the population (subgroup I, 43%; subgroup II, 46%) (Figure 2). Subgroup I is defined by a QRDR mutation in *gyrA,* with substitution of Ser83Tyr. Subgroup II isolates harbor a QRDR-associated substitution Ser83Phe along with mutations in *gyrB* and *parC*. Both subgroups were present throughout the study period. Occurrences of double and triple QRDR-associated mutations were comparatively higher in subgroup II than in subgroup I. No significant difference in the MDR phenotypes was noted between the two 4.3.1 subgroups. Subgroup I isolates differed from each other by a range of 1 to 25 SNPs, while subgroup II isolates differed by a range of 3 to 33 SNPs.

### Phenotypic and Genotypic Characterization of Antimicrobial Resistance in the Vellore Isolates

**Figure 3.**
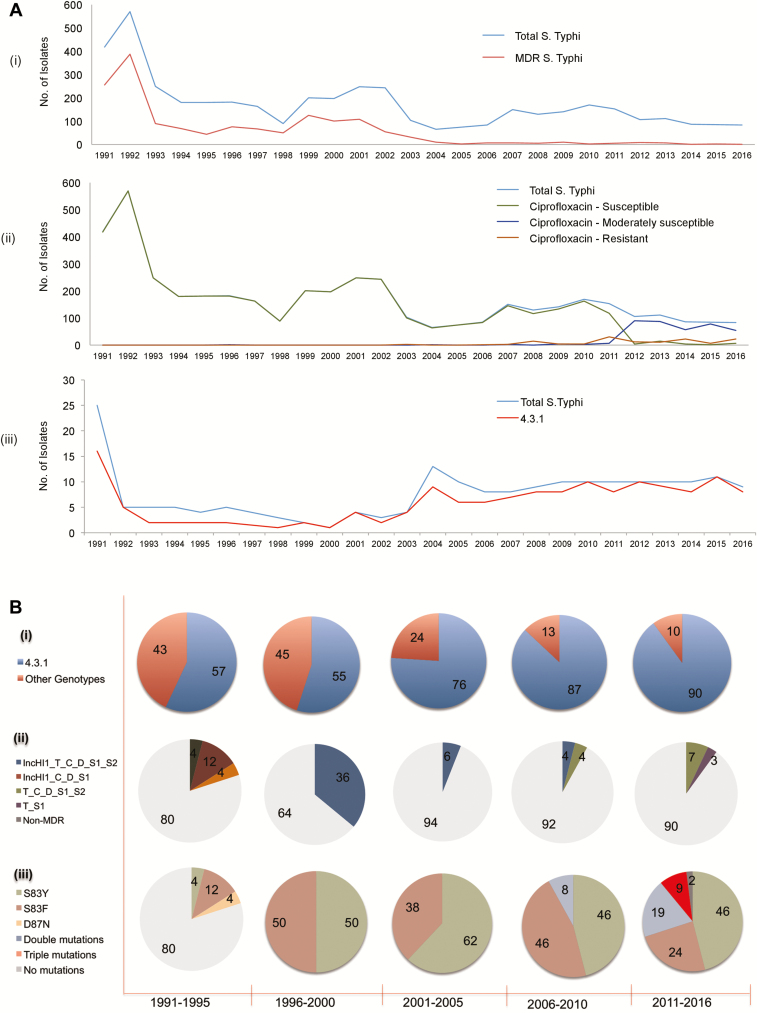
*A*, Trend analysis of typhoid fever over a 26-year period from 1991 to 2016: (i) Rates of total isolation of *Salmonella* Typhi and MDR rates, (ii) analysis of decreased ciprofloxacin susceptibility profile, and (iii) proportion of 4.3.1 genotypes among the study isolates over 26 years. *B*, Molecular characteristics of Vellore isolates of *S*. Typhi over a period 26 years during 1991 to 2016. (i) Proportion of 4.3.1 and other genotypes over the years, (ii) patterns of MDR profile observed among 4.3.1 genotypes, and (iii) mutations in quinolone resistance-determining regions of *gyrA*, *gyrB*, *parC*, and *parE* observed among 4.3.1 genotypes of Vellore isolates. The percentage of each profile is depicted. Higher MDR rates presented between 1996 and 2000 are due to fewer numbers of isolates (4 of 11 isolates). Abbreviations: MDR, multidrug resistance.

Over the 26-year study period, a total of 4462 *S*. Typhi were isolated from blood and bone marrow cultures, of which 24% (n = 1526) were MDR (resistant to ampicillin, chloramphenicol, and co-trimoxazole). The highest MDR rates were observed during 1991 to 2002, and ranged from 68% to 23%. Notably, the numbers of *S*. Typhi isolated have declined from 243 to 100 during 2002 and 2003 with MDR rates of 23% and 31%, respectively. Following this, MDR rates have been declining from 14% to 1% between 2004 and 2016, respectively. Since 2003, with the decline in MDR rates, fluoroquinolone nonsusceptibility rates (DCS) have begun to increase. This aligns with the change in the antibiotic regimen being followed for typhoid management (ie, reduction in the use of first line agents [ampicillin, chloramphenicol, and co-trimoxazole] and the increased use of fluoroquinolone). In addition, from 2011, fluoroquinolone resistance was observed to increase in addition to DCS phenotype, which was due to the high use of fluoroquinolone for typhoid management. Figure 3A summarizes the trend in typhoid fever in Vellore with MDR and DCS rates observed over 26 years. From the overall *S*. Typhi collection, isolates that could be retrieved from the archived stocks were chosen and temporal representatives distributed throughout the study period were sequenced.

Of the 194 *S*. Typhi isolates from Vellore that were sequenced, 14% (n = 21) were phenotypically and genotypically MDR. All of the MDR isolates were genotyped as 4.3.1, which is concordant with the MDR association of the 4.3.1 clade. However, 86% (n = 128) of the Vellore 4.3.1 collection was non-MDR, of which 82% (n = 105) displayed a DCS phenotype. While the MDR phenotype was not observed in other genotypes of *S*. Typhi from Vellore, 27% of these isolates also displayed a DCS profile.

Antibiotic resistance–associated genes that contribute to the MDR phenotype include *bla*_*TEM*_, *cat*, *dfrA_1*, *sul (1 and 2).* These confer resistance to ampicillin, chloramphenicol, trimethoprim and co-trimoxazole, respectively, and were consistently found in all but one of the MDR 4.3.1 isolates from our study. The results of phenotypic and molecular antibiotic resistance profiling are described in Table 1. Ampicillin and chloramphenicol resistance was correlated with the presence of *bla*_TEM_ and *cat* genes in 90% and 15% of the isolates, respectively. Trimethoprim/sulfamethoxazole-resistant isolates (96%) harbored either *dfr*A or *sul*1/2 or both genes. For ciprofloxacin resistance, 100% concordance was noted with the presence of QRDR mutations in *gyrA*, *gyrB*, *parC*, and *parE* genes. In the majority of isolates from before 2000 the MDR-associated antibiotic determinants were located on an IncHI1 plasmid. However, 8 MDR *S*. Typhi isolated after 2000 lacked IncHI1 plasmid and harbored resistance determinants integrated into the chromosome of the *S*. Typhi genome, as described previously [16, 22]. Figure 3B shows the genotypes and AMR profile in the Vellore *S*. Typhi isolates.

**Table 1.** Antimicrobial Resistance Determinants in Multidrug-Resistant 4.3.1 *Salmonella* Typhi

Name	Year of Isolation	Phenotypic Susceptibility by Disk Diffusion			Identified Based on WGS	
		AMP	CHL	SXT	AMR Genes	Plasmids
B6951	1991	R	*…*	R	*TEM, cat, dfrA_1, Sul1, Sul2*	*IncHI1*
B2033	1991	R	S	R	*…*	*IncHI1*
B3309	1991	R	R	R	*cat, dfrA_1, Sul1*	*IncHI1*
B4016	1991	R	R	R	*TEM, cat, dfrA_1, Sul1, Sul2*	*IncHI1*
B3474	1991	R	R	R	*TEM, cat, dfrA_1, Sul1, Sul2*	*IncHI1*
B4812	1991	R	R	R	*TEM, cat, dfrA_1, Sul1, Sul2*	*IncHI1*
B4636	1991	R	R	R	*TEM, cat, dfrA_1, Sul1, Sul2*	*…*
B4760	1991	R	R	R	*TEM, cat, dfrA_1, Sul1, Sul2*	*IncHI1*
B8880	1992	R	S	R	*TEM, cat, dfrA_1, Sul1, Sul2*	*IncHI1*
B8794	1992	R	S	R	*TEM, cat, dfrA_1, Sul1, Sul2*	*IncHI1*
B3848	1992	R	S	R	*TEM, cat, dfrA_1, Sul1, Sul2*	*…*
B6892	1993	R	R	R	*TEM, cat, dfrA_1, Sul1, Sul2*	*…*
B8746	1995	R	S	R	*TEM, cat, dfrA_1, Sul1, Sul2*	*IncHI1*
B8927	1996	R	R	R	*TEM, cat, dfrA_1, Sul1, Sul2*	*IncHI1*
B6327	1998	R	R	R	*TEM, cat, dfrA_1, Sul1, Sul2*	*IncHI1*
B8340	1999	R	R	R	*TEM, cat, dfrA_1, Sul1, Sul2*	*IncHI1*
B95	2000	R	R	R	*TEM, cat, dfrA_1, Sul1, Sul2*	*IncHI1*
B1781	2001	I	I	R	*TEM, cat, dfrA_1, Sul1, Sul2*	*IncHI1*
B3273	2001	R	R	R	*…*	*…*
B8541	2005	S	S	R	*TEM, cat, dfrA_1, Sul1, Sul2*	*IncHI1*
B4595	2006	R	R	R	*TEM, cat, dfrA_1, Sul1, Sul2*	*IncHI1*
B12730	2007	R	R	R	*TEM, cat, dfrA_1, Sul1, Sul2*	*…*
B5606	2009	R	R	R	*TEM, cat, dfrA_1, Sul1, Sul2*	*IncHI1*
B6855	2009	R	R	R	*TEM, cat, dfrA_1, Sul1, Sul2*	*…*
B32375	2011	R	R	R	*TEM, cat, dfrA_1, Sul1, Sul2*	*…*
B39348	2012	R	R	R	*TEM, cat, dfrA_1, Sul1, Sul2*	*…*
B24112	2012	R	R	R	*TEM, cat, dfrA_1, Sul1, Sul2*	*…*
B4129	2014	R	S	S	*TEM, Sul1*	*…*
B4878	2014	R	S	S	*TEM, Sul1*	*…*
B5087	2015	R	R	R	*TEM, cat, dfrA_1, Sul1, Sul2*	*…*

Abbreviations: AMP, ampicillin; AMR, antimicrobial resistance; CHL, chloramphenicol; I, intermediate; R, resistance; S, susceptible; SXT, trimethoprim/sulfamethoxazole; WGS, whole-genome sequencing.

**Figure 4.**
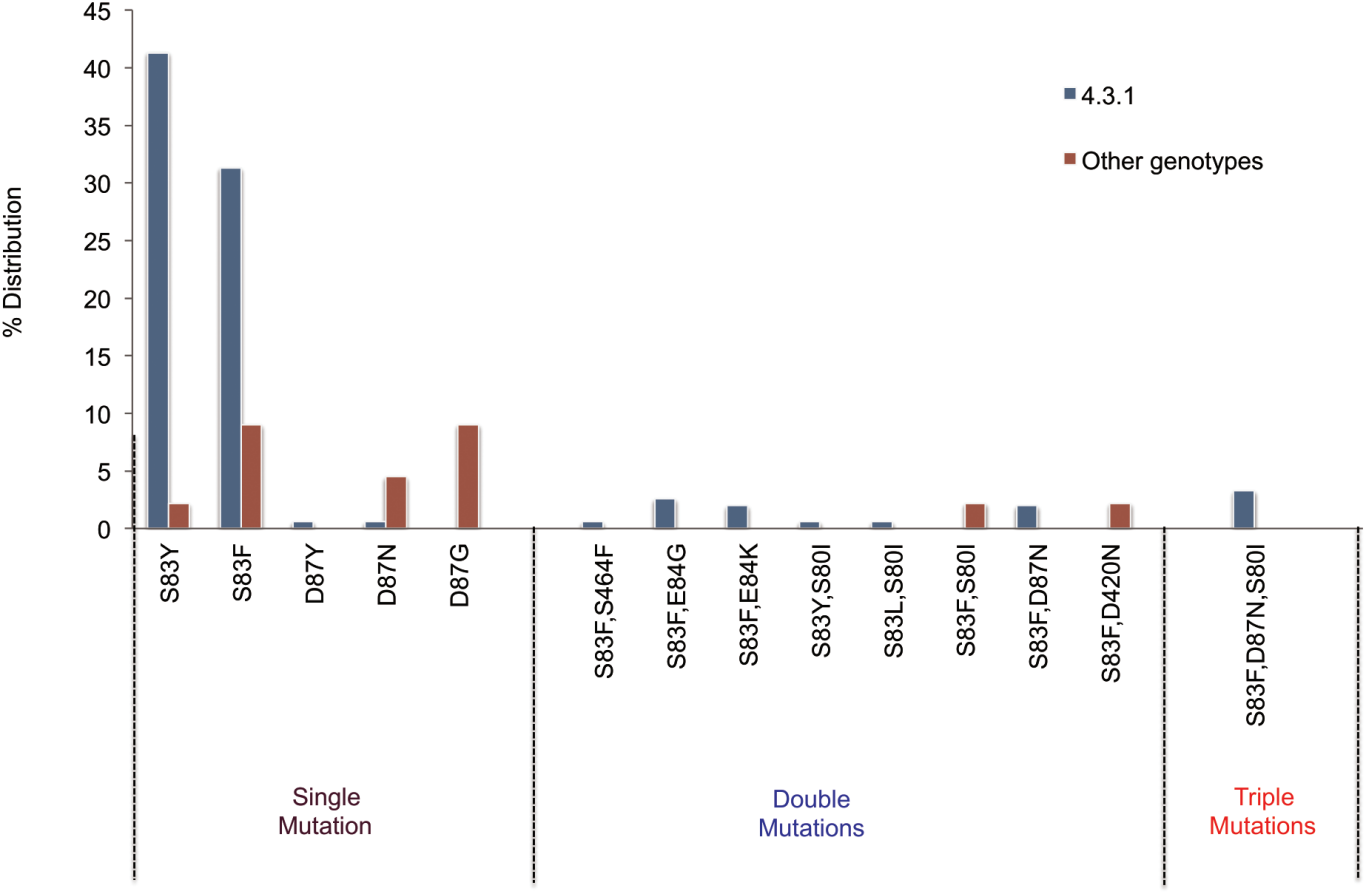
Quinolone resistance-determining region mutation profile seen in fluoroquinolone nonsusceptible *Salmonella* Typhi isolates of 4.3.1 and other genotypes identified in this study.

In terms of mutations within the QRDR, 87% (n = 129/149) of 4.3.1 and 30% (n = 13/44) of non-4.3.1 isolates harbored at least 1 mutation known to contribute to fluoroquinolone resistance. Among the 4.3.1 clade, 86% (n = 111/129) of isolates had a single QRDR-associated mutation, with 10% (n = 13/129) carrying double mutations and 4% (n = 5/129) carrying triple mutations. Most mutations were in *gyrA* (cS83Y, S83F, D87N, D87Y) and accounted for 74% of the total, followed by combinations of *gyrA* (S83F, S83Y, S83L, D87N) and *parC* (E84G, E84K,S80I) (9%). Only 1 isolate, B8784 from 2010, had a combination of *gyrA* (S83F) and *gyrB* (S464F) mutations. Similarly, *gyrA* mutations accounted for 85% of the DCS in the non-4.3.1 isolates, followed by combinations of *gyrA* + *parC* and *gyrA* + *parE* (15%). Notably, 3 QRDR mutations were detected exclusively in non-4.3.1 DCS isolates. These were D87G in *gyrA*, S83F + S80I in *gyrA* + *parC*, and S83F + 420N in *gyrA* + *parE*, respectively. QRDR-associated mutations identified in this study isolates are shown in Figure 4. One hundred percent concordance was observed for phenotypic DCS profile with QRDR mutations.

Subset analysis revealed the impact of the number of sequence-identified QRDR-associated mutations against the phenotypically determined ciprofloxacin MIC. For isolates with a single mutation within the QRDR in gyrase, ciprofloxacin MIC ranged from 0.25 to 0.38 µg/mL. Isolates with double/triple mutations within *gyrA/B* or with *parC* mutations displayed high-level MICs of 12 to 32 µg/mL or more, respectively. Among 4.3.1 isolates, the DCS phenotype became more common from the mid-1990s onward and expanded rapidly before appearing in non-4.3.1 genotypes, which were isolated after 2004.

## DISCUSSION

In this study we report the longer term dominance of the MDR-associated 4.3.1 lineage of *S*. Typhi in a region of southern India (Vellore), based on whole-genome analysis of 194 isolates collected between 1991 and 2016. Significantly, 64% (16/25) of the *S. Typhi* from the year 1991 were 4.3.1, which confirms the dominance of the 4.3.1 clade since as early as 1990s. This corrects the previous reporting of the first Indian 4.3.1 in 2004 [17].

Our study also indicates that the 4.3.1 lineage might have been circulating in this part of India before the 1990s because more than 50% of the *S*. Typhi analyzed from 1991 onward were of this genotype. In comparison with the overall global 4.3.1 clade, the Vellore isolates formed 2 distinct subgroups within the collection. Subgroup I of 4.3.1 isolates from the Vellore cluster with *S*. Typhi from countries adjacent to India in the South Asia region including Nepal, Bangladesh, and Pakistan, indicates significant regional spread of this cluster. While subgroup I isolates were obtained across these South Asian countries throughout the period of this study, indicating their continued endemicity in the region [17], the isolates from subgroup II had a broader geographical distribution, spanning Southeast Asian countries. This correlates with the fact that 4.3.1 isolates from Vietnam and Cambodia obtained over a similar timeline harbor the Ser83Phe substitution in *gyrA,* similar to subgroup II [23–25]. Acquisition of an additional Asp420Asn substitution in *parE* was associated with the localized expansion of this lineage in Vietnam [17].

Until 2000, ampicillin, chloramphenicol, and trimethoprim/sulfamethoxazole were broadly the antibiotics of choice for the management of typhoid fever. However, due to the emergence of resistance, the use of second-line agents such as fluoroquinolone, ceftriaxone, and azithromycin has been recommended. In India, due to the availability of oral formulations, fluoroquinolone usage is far greater than that for other antibiotics. This likely led to the emergence of the “DCS” phenotype, further limiting the clinical options available [22]. Effective treatment still remains a major challenge in this scenario, especially for 4.3.1 MDR *S*. Typhi with fluoroquinolone resistance, leaving ceftriaxone and azithromycin as the last-resort options [26, 27].

Fluoroquinolone resistance was also observed in non-4.3.1 *S*. Typhi, which may have also been influenced by the use of fluoroquinolone in India. The MDR *S*. Typhi of the 4.3.1 lineage was reported to be high in other parts of Asia, although we observed less than 20% [28–30]. Unusually, 4.3.1 dominance was detected in the early and later periods of this study, MDR in 4.3.1 disappeared in the mid 2000s, and very few isolates with MDR (one each in 2005, 2006, and 2009) and the IncHI1 plasmid were observed in the past few years. With these declining MDR rates, however, there has been a rise in the resistance rates to fluoroquinolone [10, 27, 31]. Further, the overall isolation of *S*. Typhi in India has been decreasing in the last few years as documented by a systematic review capturing data from 3 large tertiary-care hospitals [32, 33]. This declining trend may be a result of increasing per capita income, improved access to healthcare, early antibiotic administration, or other factors. A reduction in the population living in slums (from 55% in 1990 to 24% in 2015) has also been linked to the decline of *S*. Typhi rates in India [33].

Our study provides insights into the population of *S*. Typhi circulating in the southern India region over the past 26 years. Although the incidence of MDR *S*. Typhi, at present, is relatively low compared with that found in 4.3.1 from Southeast Asia, it is still linked to the 4.3.1 genotyope with phenotypic and genotypic evidence for high rates of fluoroquinolone resistance found in all genotypes. The 4.3.1 clade in Vellore is still the major lineage in the region today and poses a serious public health concern. The extended genotypic framework provided through our study would facilitate informed tracking of the transmission and persistence of this extraordinarily successful clade within and outside the district, state, and national boundaries [20]. Any 4.3.1 or other genotypes of *S*. Typhi isolated in the region or across India in the near future, when sequenced and added to this framework spanning the most extensive Indian *S*. Typhi to date, would guide the public health sector and clinicians alike in any change in trend. If an India-specific clade is recognized, the genomic changes synonymous with the clade will fuel the development of new molecular diagnostics for the rapid detection of not just *S. Typhi* in general but *S*. Typhi of major concern to the country. This added layer of risk attribution to the detection methodology will allow the local, national, and international public health agencies to make scientifically driven, robust decisions in their intervention and control programs. The changing trend of AMR in this lineage would enable a more targeted approach to the clinical use of antibiotics for treating typhoid fever.

## Supplementary Data

Supplementary materials are available at *Clinical Infectious Diseases* online. Consisting of data provided by the authors to benefit the reader, the posted materials are not copyedited and are the sole responsibility of the authors, so questions or comments should be addressed to the corresponding author.

ciz1112_suppl_Supplementary_Table_1

## Notes

***Acknowledgments.*** This study was approved by the Institutional Review Board (IRB) of Christian Medical College, Vellore, IRB number 10393, dated 30 November 2016

***Financial support.*** This work was supported by The Wellcome Trust (STRATAA), The Gates Foundation (TyVac), the Department of Biotechnology, and the National Institute of Health Research Cambridge Biomedical Research Council funding. A. K. P was supported for training by a grant from the Global Infectious Diseases Research Training Program, Funding Fogatry Program (project number 5D43TW007392-12; to G. K.).

***Potential conflicts of interest.*** The authors: No reported conflicts of interest. All authors have submitted the ICMJE Form for Disclosure of Potential Conflicts of Interest.
